# An Efficient and Improved Methodology for the Screening of Industrially Valuable Xylano-Pectino-Cellulolytic Microbes

**DOI:** 10.1155/2015/725281

**Published:** 2015-01-26

**Authors:** Avtar Singh, Amanjot Kaur, Anita Dua, Ritu Mahajan

**Affiliations:** ^1^Department of Biotechnology, Kurukshetra University, Kurukshetra 136 119, India; ^2^Department of Biochemistry, University College, Kurukshetra University, Kurukshetra 136 119, India

## Abstract

Xylano-pectino-cellulolytic enzymes are valuable enzymes of the industrial sector. In our earlier study, we have reported a novel and cost effective methodology for the qualitative screening of cellulase-free xylano-pectinolytic microorganisms by replacing the commercial, highly expensive substrates with agricultural residues, but the microorganisms with xylanolytic, pectinolytic, cellulolytic, xylano-pectinolytic, xylano-cellulolytic, pectino-cellulolytic, and xylano-pectino-cellulolytic potential were obtained. The probability of getting the desired combination was low, so efforts were made to further improve this cost effective methodology for obtaining the high yield of the microbes capable of producing desired combination of enzymes. By inclusion of multiple enrichment steps in sequence, using only practically low cost substrates and without any nutrient media till primary screening stage, this improved novel protocol for screening gave only the desired microorganisms with xylano-pectino-cellulolytic activity. Using this rapid, efficient, cost effective, and improved methodology, microbes with required combination of enzymes can be obtained and the probability of getting the desired microorganisms is cent percent. This is the first report presenting the methodology for the isolation of xylano-pectino-cellulolytic positive microorganisms at low cost and consuming less time.

## 1. Introduction

Lignocellulose, the most abundant natural biopolymer on earth, is an important source for the production of various industrially useful materials. Enzymatic and chemical methods can be used for the degradation of these materials. Chemical methods are performed at high temperatures and alkaline conditions, which produce toxic by-products as compared to the enzymatic methods. Chemical degradation of these materials is toxic to the environment which can be replaced by enzymatic processes or can be merged with enzymatic methods so as to reduce the concentration of toxic chemicals being used conventionally in chemical processes. Various industrial processes involving the use of microbial enzymes are less polluting, highly efficient, and energy saving and also result in lower disposal problems [1, 2]. Replacement of toxic chemicals by microbial enzymes in different industries is one of the most important fields of research these days. Enzymes which are being used extensively in various industrial processes are xylanases, pectinases, cellulases, lipases, proteases, and lignolytic enzymes. Thus, there is an increasing demand for isolating microorganisms capable of producing valuable enzymes in combination so that they could make the process cost effective at the industrial scale.

Xylano-pectino-cellulolytic enzymes are the industrially important enzymes which specifically degrade the xylan, pectin, and cellulose into sugars and are being used for the extraction of vegetable oil; processing of animal feed, food, and beverages; recycling of waste paper; textile industry; and biofuel production [2–5]. Till date, there are only few reports of their production in combination. Many workers have used the expensive substrates such as xylan [6, 7], pectin [8, 9], and cellulose [10, 11] for the screening of xylanase, pectinase, and cellulase producing microorganisms, respectively. The use of these substrates is very expensive for qualitative estimation of enzymes activity during screening process. Our earlier study replaced the use of highly expensive substrates by agricultural wastes for screening purposes, but we got both positive and negative enzymes producing microorganisms [12], while the present improved methodology gave only the desired xylano-pectino-cellulolytic positive microorganisms. Production of combination of various industrially important enzymes from a microbial isolate simultaneously in the same production medium will reduce the production cost and ultimately will help in making the process commercially viable.

## 2. Materials and Methods

### 2.1. Enrichment

Soil is the good source of microorganisms due to the nutrient rich environments, where there is a high proliferation of microorganisms. Soil samples contaminated with the effluents of various paper and textile industries were collected. These were mixed in equal proportions to make the composite samples. Enrichment step was carried out using agricultural wastes and no other nutrient medium was used. Before use, dried green citrus peel and wheat bran were washed separately and dried at 45°C and citrus peel was grinded. One gram of the composite soil sample was suspended in 100 mL Erlenmeyer flask containing 20 mL sterile deionized water, pH 10.0, and kept on shaker set at 50°C, 200 rpm for 1 h in order to uniformly mix the soil sample and centrifuged after 1 h so as to get the clear suspension. Ten percent of the above suspension was added to 20 mL sterile water (pH 10.0) supplemented with only 2% wheat bran and was incubated at 50°C, 200 rpm for 24 h. Wheat bran was used to stimulate the growth of xylanase producing microorganisms. After 24 h of incubation, 10% inoculum from wheat bran enriched culture was used to inoculate another 100 mL Erlenmeyer flask containing 20 mL sterile water (pH 10.0) supplemented with only 2% citrus peel for the enrichment of pectinase producing microorganisms and was incubated at 50°C, 200 rpm for 24 h. After incubation, 10% inoculum from wheat bran-citrus peel enriched culture was used to inoculate another 100 mL Erlenmeyer flask containing 20 mL sterile water (pH 10.0) supplemented with only 2% waste paper and was incubated at 50°C, 200 rpm for 24 h. Waste paper was used to stimulate the growth of cellulase producing microorganisms. After enrichment of xylano-pectino-cellulolytic microorganisms one by one in wheat bran, citrus peel, and waste paper, final enrichment step was carried out using all these substrates (1% each), in 20 mL sterile water (pH 10.0), and the incubation was done under the same conditions as mentioned above. This final multistep enriched culture sample was used for further study.

### 2.2. Primary Screening:* Spreading Plate Method*

For the isolation of xylano-pectino-cellulolytic microorganisms, primary screening was carried out on agar plates containing wheat bran, citrus peel, and waste paper individually and no other nutrient medium was used. Different dilutions of the enriched culture sample were plated onto agar plates containing 2% wheat bran (pH 10.0) for the screening of xylanolytic microorganisms and the plates were incubated at 50°C for 24 h. In order to evaluate the efficiency of this new methodology, different isolates having large colony size were selected from the above plates and growth was also checked onto agar plates containing 2% citrus peel and 2% waste paper individually using the same conditions as above for the screening of pectinolytic and cellulolytic microorganisms, respectively.

### 2.3. Secondary Screening:* Qualitative Analysis*

Different isolates selected in primary screening were spotted onto the agar plates containing only 0.5% peptone and 1.0% wheat bran (pH 9.0) and were incubated at 50°C for 24 h. After 24 h of incubation, xylanase producing microorganisms were selected by flooding the plates with 0.5% (w/v) Congo red for 15 min followed by repeated washing with 1 M NaCl for analyzing the zones of substrate hydrolysis [6]. Isolates which had shown clear zone on wheat bran agar plates were further spotted onto the agar plates containing only 0.5% peptone and 1.0% citrus peel (pH 9.0) and were incubated at 50°C for 24 h. After 24 h of incubation, plates were flooded with iodine solution in order to isolate the pectinase producers [13]. Pectinase producing strains were selected by analyzing the substrate clearance zone. Strains which had shown zone on wheat bran and citrus peel agar plates were further spotted onto the agar plates containing only 0.5% peptone and 1.0% waste paper (pH 9.0) and were incubated under the same conditions as given above. After incubation, cellulase producing microorganisms were selected by flooding the plates with 0.5% (w/v) Congo red for 15 min followed by repeated washing with 1 M NaCl. To determine the efficacy of these agricultural wastes, strains were also spotted onto the agar plates containing 0.5% peptone and 0.25% commercial xylan, pectin, and cellulose individually and zones of substrate hydrolysis were analyzed as given above.

### 2.4. Final Screening:* Quantitative Analysis*

Final screening was done by quantitative estimation of enzymes activity after producing them under submerged fermentation.

#### 2.4.1. Enzymes Production

To 20 mL sterile water containing 0.5% peptone, we added 2% wheat bran, 2% citrus peel, and 2% wheat straw, pH 8.0, for simultaneous production of xylanase, pectinase, and cellulase enzymes, respectively. In this study, yeast extract, KNO_3_, KH_2_PO_4_, MgSO_4_, or any other additive has also not been added in the medium, as described in our earlier study [12]. The media flasks were inoculated with 2% of 18-hour-old culture and were incubated at 37°C for 24 h on shaker set at 200 rpm. The extracellular enzymes were harvested by centrifuging at 10,000 g for 10 min and the clear supernatant was used for the estimation of enzymes activity.

#### 2.4.2. Enzymes Activity Analysis

Birchwood xylan 1%, pectin 0.5%, and carboxymethyl cellulose 1% were used for estimation of xylanase, pectinase, and cellulase activity, respectively. The enzymes activity was determined by measuring the amount of reducing sugars liberated after enzyme substrate reaction using 3,5-dinitrosalicylic acid reagent [14]. The reaction mixture for each enzyme assay contained 490 *μ*L of respective substrate (prepared in glycine-NaOH buffer, pH 8.5) and 10 *μ*L of appropriately diluted enzyme and was incubated at 55°C for 10 min. One unit of xylanase, pectinase, and cellulase activity is defined as the amount of enzyme that catalyzes the release of 1 nanomole of xylose, galacturonic acid, and glucose, respectively, per second under the specified assay conditions.

## 3. Results and Discussion

Due to the high cost of commercial substrates for screening purposes, the alternative cost effective agricultural wastes were used in our earlier study for the isolation of xylano-pectinolytic microorganisms [12]. In the present study, multiple enrichment steps have been included to isolate the microorganisms capable of producing desired combination of enzymes. The pH and temperature were kept high during different screening steps in order to isolate the alkalothermophilic microorganisms. The novelty of this paper is that the microorganisms obtained after enrichment and primary screening steps showed growth on all the agricultural wastes used and hence commercial medium was completely replaced with agricultural residues till primary screening stage and only commercial peptone was added to agroresidues in the secondary screening stage.

**Figure 1:**
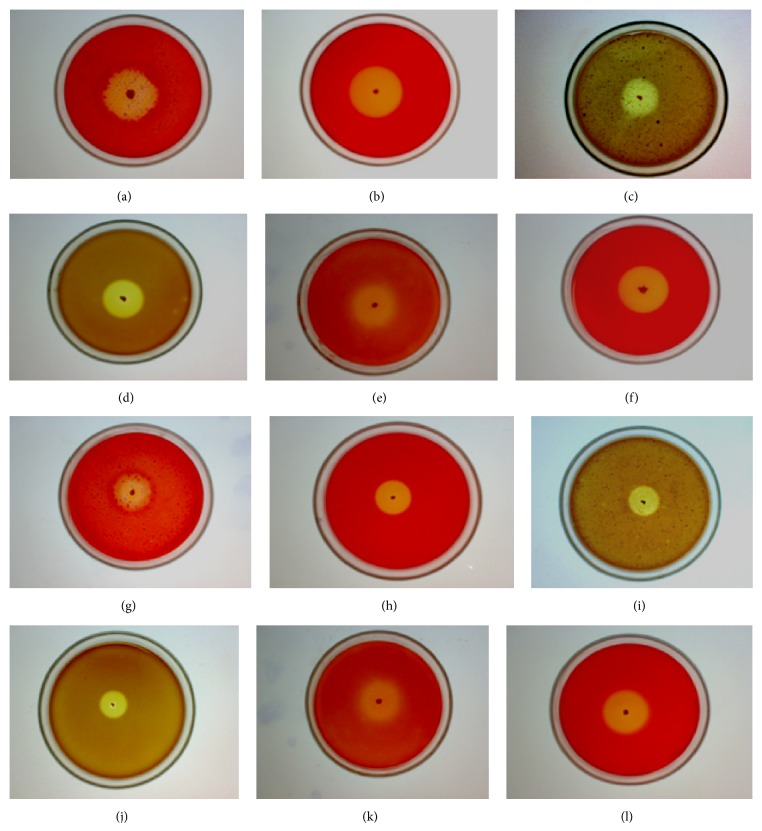
Zone of substrate hydrolysis shown on (a) wheat bran; (b) xylan; (c) citrus peel; (d) pectin; (e) waste paper; (f) CMC by xylano-pectino-cellulolytic bacterial isolate AVS13 and zone of substrate hydrolysis produced by a known xylanase producer* Bacillus stearothermophilus* on (g) wheat bran, (h) xylan; a known pectinase producer* Bacillus subtilis* on (i) citrus peel, (j) pectin; and a known cellulase producer* Flavobacterium bolustinum* on (k) waste paper, (l) CMC.

In qualitative analysis, only 0.5% peptone was added to agar plates containing wheat bran, citrus peel, and waste paper individually. Formation of large clear zone around the colonies indicated that the strains were enzymes producer with good substrate hydrolyzing ability. Zone of substrate hydrolysis obtained after Congo red staining on peptone-wheat bran agar plates indicated the xylanolytic nature of the microorganisms. In order to check the efficiency of this methodology, zones were also analysed on peptone agar plate containing commercial xylan. The clearance zones of substrate hydrolysis shown by isolate on peptone agar plate containing wheat bran/xylan (Figures 1(a) and 1(b)) were nearly similar. The highly expensive commercial xylan has been used by several workers [6, 7, 15–17] for the screening of xylanolytic microorganisms. Isolates which showed clear zone of substrate hydrolysis on wheat bran containing plates also gave the substrate clearance zone onto peptone agar medium containing citrus peel, thus indicating the pectinolytic nature of microorganisms. Similarly, comparable zones of substrate hydrolysis by the isolate on peptone agar plate containing citrus peel (Figure 1(c)) or commercial pectin (Figure 1(d)) were obtained. Many workers have reported the use of pectin for qualitative screening of pectinase producing microorganisms [8, 9, 18–20]. Isolates which showed clear zone on wheat bran and citrus peel containing plates were transferred onto waste paper containing plate. The cellulase producing microorganisms also showed zones of substrate hydrolysis of nearly the same size on both peptone-waste paper agar medium (Figure 1(e)) and peptone-cellulose agar medium (Figure 1(f)). Cellulose degrading microbes have been screened by many workers using cellulose/CM-cellulose [10, 21–23].

Table 1 shows that diameters of zone of substrate hydrolysis on agricultural wastes and commercial substrates were nearly similar. The xylano-pectino-cellulolytic bacterial isolate AVS13 screened on these agricultural wastes produced 368 ± 36, 301 ± 22, and 100 ± 16 nkat/mL of xylanase, pectinase, and cellulase, respectively, under unoptimized conditions using agricultural wastes, low amount of peptone, and no additive/enhancer of these enzymes was added in the fermentation medium (Table 1). This isolate has been identified as* Bacillus subtilis* on the basis of morphological, physiological, and biochemical tests and 16S rDNA sequencing and was found to be alkalothermophilic (Table 2). The 16S rDNA sequence has been deposited to the accessible database, NCBI GenBank, with the accession number KM110978. A known xylanase producer* Bacillus stearothermophilus* SDX (MTCC 8508), a cellulase producer* Flavobacterium bolustinum* (MTCC 10203), and a pectinase producer* Bacillus subtilis* SS (MTCC 8509) were used as control microorganisms in this study, in order to validate the efficacy of these agricultural wastes for the isolation of xylano-pectino-cellulolytic microorganisms. These control microorganisms also produced the substrate clearance zones of the same size on nutrient-agar plates containing commercial substrates and agricultural wastes separately (Figures 1(g)–1(l)).

In our earlier study, we got both positive and negative desired enzymes producing microorganisms [12], while, in this improved study, we obtained only the desired xylano-pectino-cellulolytic positive microorganisms due to inclusion of multiple enrichment steps during isolation. Similarly, this protocol was used for the isolation of acidophilic and neutrophilic microorganisms producing xylano-pectino-cellulolytic enzymes by setting the pH of the medium in the lower and neutral range and by collecting suitable composite soil sample from fruit juice and animal feed industry for screening purposes, as these enzymes are also being used in the processing of fruit juices, oil extraction, animal feed industry, and so forth (data not shown).

## 4. Conclusion

Concurrent production of these industrially important enzymes from a microbial isolate simultaneously in the same production medium will reduce the production cost, maintenance cost, man power, and so forth, to a higher extent in comparison to production of individual enzymes. This novel, improved, and efficient protocol for the screening of microorganisms gave only the desired xylano-pectino-cellulolytic positive microorganisms. Using this methodology, probability of getting the desired microorganisms is high, so this methodology would definitely save time and cost for getting the required microbial isolates. Enzymes producing industries are still looking for microbial isolates having combination of various enzymes, suitable to the needs of the industry. This methodology would also definitely help the industries in the isolation of microbes capable of producing multiple enzymes with high titre at practically low cost.

## Figures and Tables

**Table 1:** Zones of substrate hydrolysis and activity analysis of xylano-pectino-cellulolytic enzymes.

Isolates	Qualitative analysis						Quantitative analysis		
	Diameter (mm) of zone of substrate hydrolysis on agricultural wastes			Diameter (mm) of zone of substrate hydrolysis on commercial substrates			Enzyme activity nkat/mL^a^		
	Wheat bran	Citrus peel	Waste paper	Xylan	Pectin	Cellulose	Xylanase	Pectinase	Cellulase
AVS 1	27 ± 1	20 ± 1	5 ± 1	28 ± 1	21 ± 1	5 ± 1	167 ± 28	134 ± 17	3.5 ± 0.5
AVS 2	22 ± 1	20 ± 2	13 ± 1	22 ± 1	20 ± 1	14 ± 3	83 ± 16	117 ± 13	22 ± 1.5
AVS 3	31 ± 1	14 ± 1	5 ± 1	32 ± 1	15 ± 1	5 ± 1	233 ± 35	50 ± 8	3.2 ± 0.6
AVS 4	20 ± 2	23 ± 2	18 ± 1	20 ± 1	24 ± 2	18 ± 1	69 ± 8	184 ± 26	30 ± 3.5
AVS 5	25 ± 1	19 ± 1	25 ± 1	26 ± 1	19 ± 1	26 ± 1	150 ± 18	101 ± 18	42 ± 4
AVS 6	24 ± 1	14 ± 1	29 ± 2	24 ± 1	14 ± 1	30 ± 3	117 ± 14	50 ± 8	54 ± 5.2
AVS 7	32 ± 1	13 ± 1	26 ± 1	33 ± 1	13 ± 1	26 ± 1	267 ± 30	25 ± 5	41 ± 5
AVS 8	38 ± 1	11 ± 1	6 ± 1	39 ± 2	11 ± 1	6 ± 1	852 ± 62	20 ± 3.5	5 ± 0.8
AVS 9	28 ± 1	14 ± 1	10 ± 1	28 ± 1	13 ± 1	10 ± 1	184 ± 31	35 ± 4	17 ± 1
AVS 10	18 ± 2	16 ± 1	7 ± 1	17 ± 1	16 ± 1	7 ± 1	35 ± 7	67 ± 9	8.5 ± 0.8
AVS 11	17 ± 1	29 ± 3	12 ± 1	17 ± 1	30 ± 3	12 ± 1	25 ± 3.5	434 ± 41	20 ± 1.5
AVS 12	14 ± 1	11 ± 1	8 ± 1	14 ± 1	10 ± 1	8 ± 1	9 ± 1.5	17 ± 2.5	11 ± 1
AVS 13	**34 ± 1**	**26 ± 2**	**32 ± 2**	**35 ± 1**	**26 ± 2**	**32 ± 1**	**368 ± 36**	**301 ± 22**	**100 ± 16**
AVS 14	19 ± 2	23 ± 1	22 ± 1	19 ± 1	24 ± 1	24 ± 1	57 ± 11	167 ± 18	40 ± 4
AVS 15	32 ± 1	18 ± 1	11 ± 1	34 ± 1	18 ± 1	10 ± 1	284 ± 17	84 ± 9	14 ± 1.2
AVS 16	32 ± 1	13 ± 1	7 ± 1	33 ± 2	13 ± 1	7 ± 1	267 ± 29	34 ± 3.5	8 ± 1
AVS 17	32 ± 1	20 ± 2	22 ± 1	33 ± 1	21 ± 2	22 ± 3	251 ± 14	117 ± 14	30 ± 3
AVS 18	20 ± 3	8 ± 1	26 ± 1	20 ± 1	9 ± 1	27 ± 1	69 ± 10	9 ± 1	45 ± 4.2
AVS 19	24 ± 1	20 ± 1	5 ± 1	24 ± 1	21 ± 1	6 ± 1	117 ± 16	104 ± 15	3.5 ± 0.5
AVS 20	30 ± 1	8 ± 1	18 ± 1	31 ± 1	9 ± 1	18 ± 1	217 ± 25	14 ± 1.5	25 ± 2.5
AVS 21	30 ± 1	13 ± 1	12 ± 1	30 ± 1	14 ± 1	12 ± 1	201 ± 17	50 ± 6	20 ± 1.5
AVS 22	34 ± 1	16 ± 1	17 ± 1	35 ± 1	16 ± 1	17 ± 1	384 ± 33	58 ± 10	27 ± 2
AVS 23	34 ± 2	13 ± 1	26 ± 1	35 ± 3	13 ± 1	27 ± 1	401 ± 28	34 ± 3.5	35 ± 3.5
AVS 24	22 ± 1	10 ± 1	14 ± 1	23 ± 1	11 ± 1	14 ± 1	84 ± 0.9	13 ± 1.5	24 ± 2.5
AVS 25	31 ± 1	12 ± 1	8 ± 1	32 ± 1	12 ± 1	8 ± 1	234 ± 20	20 ± 2	12 ± 1.2
AVS 26	30 ± 2	15 ± 1	10 ± 1	31 ± 1	16 ± 1	10 ± 1	217 ± 18	42 ± 5	14 ± 1.5
AVS 27	19 ± 1	13 ± 1	5 ± 1	20 ± 1	13 ± 1	6 ± 1	58 ± 6	27 ± 3	3.2 ± 0.8
AVS 28	34 ± 1	26 ± 3	27 ± 1	35 ± 2	28 ± 2	28 ± 1	317 ± 32	267 ± 31	44 ± 4.5
AVS 29	31 ± 1	24 ± 1	33 ± 2	32 ± 1	25 ± 2	34 ± 1	234 ± 21	217 ± 26	92 ± 13
AVS 30	30 ± 1	9 ± 1	29 ± 3	30 ± 1	8 ± 1	30 ± 2	201 ± 13	9 ± 0.8	67 ± 8.5
AVS 31	32 ± 1	24 ± 2	30 ± 1	33 ± 2	25 ± 1	30 ± 1	267 ± 27	200 ± 17	54 ± 6
AVS 32	25 ± 1	18 ± 1	7 ± 1	25 ± 1	18 ± 1	8 ± 1	134 ± 12	87 ± 8	12 ± 1
AVS 33	32 ± 2	15 ± 1	23 ± 1	32 ± 1	15 ± 1	24 ± 1	251 ± 15	39 ± 4	35 ± 3.2
AVS 34	35 ± 2	25 ± 1	5 ± 1	37 ± 1	25 ± 1	5 ± 1	585 ± 61	184 ± 17	3.5 ± 0.8
AVS 35	30 ± 1	24 ± 2	27 ± 1	31 ± 1	24 ± 3	28 ± 1	217 ± 30	201 ± 25	44 ± 4
AVS 36	17 ± 2	8 ± 1	13 ± 1	18 ± 1	8 ± 1	14 ± 1	27 ± 3.2	12 ± 1	21 ± 2
AVS 37	32 ± 1	18 ± 1	22 ± 2	32 ± 1	19 ± 1	23 ± 1	234 ± 23	100 ± 17	34 ± 3.5
AVS 38	20 ± 1	16 ± 1	15 ± 1	20 ± 1	16 ± 1	16 ± 1	67 ± 14	54 ± 8	25 ± 2
AVS 39	32 ± 1	14 ± 1	8 ± 1	32 ± 2	15 ± 1	8 ± 1	251 ± 25	35 ± 3.5	12 ± 1.2
AVS 40	32 ± 1	15 ± 2	22 ± 1	32 ± 1	15 ± 2	24 ± 1	234 ± 20	51 ± 5	36 ± 4
AVS 41	34 ± 1	8 ± 1	29 ± 2	35 ± 1	8 ± 1	30 ± 3	351 ± 33	8 ± 0.75	54 ± 5
AVS 42	36 ± 3	14 ± 1	6 ± 1	38 ± 2	14 ± 1	6 ± 1	702 ± 42	40 ± 3.5	7 ± 1.5
AVS 43	20 ± 1	24 ± 1	6 ± 1	20 ± 1	25 ± 1	6 ± 1	67 ± 16	201 ± 23	5 ± 1
AVS 44	25 ± 2	28 ± 2	8 ± 1	25 ± 1	30 ± 2	8 ± 1	134 ± 21	434 ± 37	10 ± 1.5
AVS 45	19 ± 1	26 ± 1	7 ± 1	19 ± 1	27 ± 3	7 ± 1	50 ± 7	267 ± 28	9 ± 1.2
AVS 46	32 ± 2	26 ± 1	33 ± 2	33 ± 1	27 ± 1	34 ± 2	267 ± 24	117 ± 16	82 ± 15
AVS 47	35 ± 2	14 ± 1	8 ± 1	36 ± 1	14 ± 1	8 ± 1	518 ± 38	34 ± 3.5	10 ± 1.5
AVS 48	32 ± 1	15 ± 1	13 ± 1	32 ± 1	16 ± 1	13 ± 1	251 ± 23	57 ± 5	21 ± 2
AVS 49	25 ± 1	14 ± 1	8 ± 1	25 ± 1	15 ± 1	8 ± 1	134 ± 16	50 ± 4	8.5 ± 1.2
AVS 50	30 ± 1	8 ± 1	22 ± 1	31 ± 1	8 ± 1	23 ± 2	217 ± 20	14 ± 1.5	37 ± 3.2

^a^Under unoptimized conditions.

**Table 2:** Morphological, physiological, biochemical tests and 16S rDNA sequencing of the isolated microorganism AVS 13.

Tests	Results	Tests	Results
Morphological tests:			

Colony Morphology			
Configuration	Circular	Margin	Entire
Elevation	Raised	Surface	Rough
Texture	Mucoid	Pigment	Off-white
Opacity	Opaque	Gram's Reaction	+
Cell shape	Rod	Spore(s)	+
Motility	+		

Physiological tests:			

Growth at temperatures			
4°C	−	15°C	−
25°C	+	30°C	+
37°C	+	42°C	+
55°C	+		

Growth at pH			
pH 5.0	+	pH 6.0	+
pH 7.0	+	pH 8.0	+
pH 9.0	+	pH 10.0	+
pH 11.0	+	pH 12.0	+

Growth on NaCl (%)			
2.0	+	4.0	+
6.0	+	8.0	+
10.0	+	11.0	+
12.0	+		

Biochemical tests:			

Growth on MacConkey	Non Lactose Fermenting	Indole test	−
Methyl red test	−	Voges Proskauer test	+
Citrate utilization	−	Casein hydrolysis	+
Esculin hydrolysis	+	Gelatin hydrolysis	+
Starch hydrolysis	+	Nitrate reduction	+
Ornithine decarboxylase	+	Lysine decarboxylase	+
Catalase Test	+	Oxidase test	+

Acid production from			
Adonitol	−	Trehalose	+
Rhamnose	−	Salicin	+
Dulcitol	−	Galactose	−
Melibiose	+	Raffinose	−
Inulin	+	Sorbitol	+
Fructose	+	Sucrose	+

16S rDNA SEQUENCING			

TGCAGTCGAGCGGACAGATGGGAGCTTGCTCCCTGATGTTAGCGGCGGACGGGTGAGTAAC			
ACGTGGGTAACCTGCCTGTAAGACTGGGATAACTCCGGGAAACCGGGGCTAATACCGGATG			
GTTGTTGAACCGCATGGTTCAAACATAAAAGGTGGCTTCGGCTACCACTTACAGATGGACC			
CGCGGCGCATTAGCTAGTTGGTGAGGTAACGGCTCACCAAGGCAACGATGCGTAGCCGACC			
TGAGAGGGTGTCGGCCACACTGGGACTGAGACACGGCCCAGACTCCTACGGGAGGCAGCA			
GTAGGGAATCTTCCGCAATGGACGAAAGTCTGACGGAGCAACGCCGCGTGAGTGATGAAGG			
TTTTCGGATCGTAAAGTCTGTTGTTAGGGAAGAACAAGTACCGTTCGAATAGGGCGGTACC			
TTGACGGTACCTAACCAGAAAGCCACGGCTAACTACGTGCCAGCAGCCGCGGTAATACGTA			
GGTGGCAAGCGTTGTCCGGAATATTGGGCGTAAAGGGCTCGCAGGCGGTTTCTTAAGTCTG			
ATGTGAAAGCCCCCGGCTCAACCGGGGAGGGTCATTGGAAACTGGGGAACTTGAGTGCAGA			
AGAGGAGAGTGGAATTCCACGTGTAGGGTGAAATGCGTAGAGATGTGGAGGAACACCAGT			
GGCGAAGGCGACTCTCTGGTCTGTAACTGACGCTGAGGAGCGAAAGCGTGGGGAGCGAACA			
GGATTAGATACCCTGGTAGTCCACGCCGTAAAGATGAGTGCTAAGTGTTAGGGGGTTTCCG			
CCCCTTAGTGCTGCAGCTAACGCATTAAGCACTCCGCCTGGGGAGTACGGTCGCAAGACTGA			
AACTCAAAGGAATTGACGGGGGCCCGCACAAGCGGTGAGCATGTGGTTTAATTCGAAGCA			
ACGCGAAGAACCTTACCAGGTCTTGACATCCTCTGACAATCCTAGAGAGATAGGACGTCCCC			
TTCGGGGGCAGAGTGACAGGTGGTGCATGGTTGTCGTCAGCCGTGTCGTGAGATGTTGGGT			
TAAGTCCCGCAACGAGCGCAACCCTTGATCTTAGTTGCCAGCATTCAGTTGGGCACTCTAAG			
GTGACTGCCGGTGACAAACCGGAGGAAGGTGGGGATGACGTCAAACATCATGCCCCTTAT			
GACCTGGGCTACACACGTGCTACAATGGACAGAACAAAGGGCAGCGAAACCGCGAGGTTA			
AGCCAATCCCACAAATCTGTTCTCAGTTCGGATCGCAGTCTGCAACTCGACTCGTGAAGCT			
GGAATCGCTAGTAATCGCGGATCAGCATGCCGCGGTGAATACGTTCCCGGGCCTTGTACACA			
CCGCCCGTCACACCACGAGAGTTTGTAACACCCGAAGTCGGTGAGGTAACCTTTAGG			

+: Positive; −: Negative.

## References

[1] Kaur A., Mahajan R., Singh A., Garg G., Sharma J. (2010). Application of cellulase-free xylano-pectinolytic enzymes from the same bacterial isolate in biobleaching of kraft pulp. *Bioresource Technology*.

[2] Singh A., Yadav R. D., Kaur A., Mahajan R. (2012). An ecofriendly cost effective enzymatic methodology for deinking of school waste paper. *Bioresource Technology*.

[3] Bhat M. K. (2000). Cellulases and related enzymes in biotechnology. *Biotechnology Advances*.

[4] Jayani R. S., Saxena S., Gupta R. (2005). Microbial pectinolytic enzymes: a review. *Process Biochemistry*.

[5] Polizeli M. L. T. M., Rizzatti A. C. S., Monti R., Terenzi H. F., Jorge J. A., Amorim D. S. (2005). Xylanases from fungi: properties and industrial applications. *Applied Microbiology and Biotechnology*.

[6] Gessesse A., Gashe B. A. (1997). Production of alkaline xylanase by an alkaliphilic *Bacillus* sp. isolated from an alkaline soda lake. *Journal of Applied Microbiology*.

[7] Gupta V. K., Gaur R., Gautam N., Kumar P., Yadav I. J., Darmwal N. S. (2009). Optimization of xylanase production from *Fusarium solani* F7. *The American Journal of Food Technology*.

[8] Ahlawat S., Battan B., Dhiman S. S., Sharma J., Mandhan R. P. (2007). Production of thermostable pectinase and xylanase for their potential application in bleaching of kraft pulp. *Journal of Industrial Microbiology and Biotechnology*.

[9] Jacob N., Niladevi K. N., Anisha G. S., Prema P. (2008). Hydrolysis of pectin: an enzymatic approach and its application in banana fiber processing. *Microbiological Research*.

[10] Baharuddin A. S., Razak M. N. A., Hock L. S. (2010). Isolation and characterization of thermophilic cellulase-producing bacteria from empty fruit bunches-palm oil mill effluent compost. *The American Journal of Applied Sciences*.

[11] Bakar N. K. A., Abd-Aziz S., Hassan M. A., Ghazali F. M. (2010). Isolation and selection of appropriate cellulolytic mixed microbial cultures for cellulases production from oil palm empty fruit bunch. *Biotechnology*.

[12] Kaur A., Mahajan R., Singh A., Garg G., Sharma J. (2011). A novel and cost effective methodology for qualitative screening of alkalo-thermophilic cellulase free xylano-pectinolytic microorganisms using agricultural wastes. *World Journal of Microbiology and Biotechnology*.

[13] Fernandes-Salomão T. M., Rodrigues Amorim A. C., Chaves-Alves V. M., Cavalcante Coelho J. L., Silva D. O., Fernandes De Araújo E. (1996). Isolation of pectinase hyperproducing mutants of *Penicillium expansum*. *Revista de Microbiologia*.

[14] Miller G. L. (1959). Use of dinitrosalicylic acid reagent for determination of reducing sugar. *Analytical Chemistry*.

[15] Sridevi B., Charya M. A. S. (2011). Isolation, identification and screening of potential cellulase-free Xylanase producing fungi. *African Journal of Biotechnology*.

[16] Kamble R. D., Jadhav A. R. (2012). Isolation, purification, and characterization of xylanase produced by a new species of *bacillus* in solid state fermentation. *International Journal of Microbiology*.

[17] Sanghvi G., Jivrajani M., Patel N., Jivrajani H., Bhaskara G. B., Patel S. (2014). Purification and characterization of haloalkaline, organic solvent stable xylanase from newly isolated halophilic bacterium-OKH. *International Scholarly Research Notices*.

[18] Anisa S. K., Ashwini S., Girish K. (2013). Isolation and screening of *Aspergillus* spp.for pectinolytic activity. *Electronic Journal of Biology*.

[19] Anisa S. K., Girish K. (2014). Pectinolytic activity of *Rhizopus* sp. and *Trichoderma viride*. *International Journal of Research in Pure and Applied Microbiology*.

[20] Kusuma M. P., Reddy D. S. R., Sharma M. (2014). Screening of alkalophilic and thermophilic potential isolate for production of polygalacturonase. *International Journal of Innovative Research in Science, Engineering and Technology*.

[21] Gomashe A. V., Gulhane P. A., Bezalwar P. M. (2013). Isolation and screening of cellulose degrading microbes from nagpur region soil. *International Journal of Life Sciences*.

[22] Das P., Solanki R., Khanna M. (2014). Isolation and screening of cellulolytic actinomycetes from diverse habitats. *International Journal of Advanced Biotechnology and Research*.

[23] Patagundi B. I., Shivasaran C. T., Kaliwal B. (2014). Isolation and characterization of cellulase producing bacteria from soil. *International Journal of Current Microbiology and Applied Sciences*.

